# Health related quality of life among haemodialysis and kidney transplant recipients from Nepal: a cross sectional study using WHOQOL-BREF

**DOI:** 10.1186/s12882-020-02085-0

**Published:** 2020-10-12

**Authors:** Kamal Ranabhat, Pratik Khanal, Shiva Raj Mishra, Anu Khanal, Sangita Tripathi, Mahesh Raj Sigdel

**Affiliations:** 1 Department of Health Services, Ministry of Health and Population, Kathmandu, Nepal; 2 Institute of Medicine, Tribhuvan University, Kathmandu, Nepal; 3 World Heart Federation, Geneva, Switzerland

**Keywords:** Kidney, Nepal, End stage renal disease, Quality of life, haemodialysis, transplant

## Abstract

**Background:**

Very less is known about health-related quality of life (HRQOL) among patients with kidney diseases in Nepal. This study examined HRQOL among haemodialysis and kidney transplant recipients in Nepal.

**Methods:**

The Nepali version of World Health Organization Quality of Life Instruments -(WHOQOL-BREF) questionnaire was administered using face to face interviews among end stage renal disease (ESRD) patients, from two large national referral centers in Nepal. The differences in socio-demographic characteristics among ESRD patients were examined using the Chi-square test. The group differences in quality of life (QOL) were examined using the Mann-Whitney U test and Kruskal-Wallis tests.

**Results:**

Of the 161 participants, 92 (57.1%) were renal transplant recipients and 69 (42.9%) patients were on maintenance haemodialysis. Hypertension (70.9%) was the most common co-morbidity among ESRD patients. Haemodialysis patients scored significantly lower than the transplant recipients in all four domains as well as in overall perception of quality of life and general health. Ethnicity (*p* = 0.020), socio-economic status (*p* < 0.001), educational status (p < 0.001) and employment status (*p* = 0.009) were significantly associated with the overall QOL in ESRD patients. Across patient groups, educational status (*p* = 0.012) was positively associated with QOL in dialysis patients, while urban residence (*p* = 0.023), higher socio-economic status (p < 0.001), higher educational status (*p* = 0.004) and diabetes status (*p* = 0.010) were significantly associated with better QOL in transplant recipients.

**Conclusion:**

The overall QOL of the renal transplant recipients was higher than that of the patients on maintenance haemodialysis; this was true in all four domains of the WHOQOL-BREF. ESRD patients with low HRQOL could benefit from targeted risk modification intervention.

## Background

The incidence and prevalence of end-stage renal disease (ESRD) has been rising globally, yet the burden in South Asia is not known precisely due to improper registration systems [1]. A population-based study assessed the age-adjusted incidence of ESRD at 232 cases per million populations per year in India [2]. In Nepal, the estimated incidence of ESRD is approximately 2900/year [3, 4].

ESRD is an increasingly recognized pandemic that is associated with high cost and financial burden to patients, families and the health system of any country [5–7]. In Nepal, the burden of ESRD is growing, however only a fraction of ESRD patients receive renal replacement therapy every year [8]. ESRD treatment is costly and unaffordable for most Nepalese people, although the Government of Nepal provides payment to the hospital to cover some cost for haemodialysis and transplant recipients [3, 9, 10]. Infrequent and inadequate haemodialysis along with malnutrition and frequent use of blood transfusion are some of the major problems prevailing in Nepalese haemodialysis patients [11–13]; at the same time, expenses of the post-transplant medicines and distance to travel for regular follow up apparently affect the kidney transplant recipients [9]. Naturally, the quality of life (QOL) of ESRD patients on maintenance haemodialysis and kidney transplant recipients is compromised impacting various health outcomes [14, 15].

Assessment of QOL could be a valuable research tool in assessing the outcome of therapeutic intervention in chronic diseases [16–18]. The World Health Organisation (WHO) has defined QOL as an individual’s perceptions of their position in life in the context of the culture and value systems where they live and in relation to their goals, expectations, standards and concerns [16]. Globally, health related quality of life (HRQOL) has been recognized as an important tool in the assessment of the health and wellbeing of people receiving renal replacement therapies (RRT) [19–21].

Although kidney transplantation was legalised in 2002, the first successful live donor kidney transplantation was performed in Tribhuvan University Teaching Hospital (TUTH) in 2008 [22, 23]. There are currently 42 haemodialysis and five transplant centers in Nepal for its 30 million people. All five transplant centers and 17 out of 42 haemodialysis centers are situated in the Kathmandu valley, the capital city of the country [24]. A paucity of information on the QOL among patients attending ESRD services exists in Nepal. Furthermore, limited studies are available to compare QOL in ESRD patients receiving different RRTs. In this context, this study aimed to assess and compare the QOL of haemodialysis and renal transplant recipients in various dimensions using WHOQOL BREF. We expect that the current study will support policymakers and concerned authorities in developing better interventions and programs for ESRD patients in Nepal.

## Methods

### Study settings

The study was conducted in haemodialysis patients attending TUTH and renal transplant recipients attending the National Public Health Laboratory (NPHL) under the Ministry of Health and Population (MoHP). The patients who underwent renal transplant visited NPHL for the purpose of monitoring kidney function tests and other labs as part of their follow-up. Both of these institutions are situated in Kathmandu, the capital city of Nepal.

### Study design and sampling technique

A cross-sectional comparative study design was adopted to assess the HRQOL between haemodialysis and renal transplant recipients. The NPHL was chosen for recruiting renal transplant recipients, while TUTH was chosen for recruiting haemodialysis patients for face-to-face interviews.

A total of 182 patients were approached for data collection of which 161 patients were recruited from the study sites in October and November in 2018. Coordination with the officials of the Department of Nephrology in TUTH and chronic kidney disease (CKD) service unit overseeing impoverishment citizen funds of NPHL was performed before approaching the study participants. All patients meeting the eligibility criteria during the data collection period were recruited as study participants. The eligibility for haemodialysis patients included those receiving haemodialysis at least twice a week for three months or more while for transplant recipients included those who were at least six months post kidney transplantation. Other eligibility for the study participants were those at least 18 years of age and were able to provide written consent. A total of 21 patients who had serious health complications and mental health disorders such as advanced liver failure, advanced heart failure, advanced respiratory problems, history of stroke affecting self-care and movement, and advanced dementia were excluded from the study because these conditions were expected to hamper the HRQOL of the patients. Similarly, those on peritoneal dialysis were not included since very few patients from TUTH are on peritoneal dialysis as RRT.

### Data collection measures

The questionnaire comprised three sections. These included (i) socio-demographic information; (ii) information related to kidney disease and (iii) HRQOL of the study participants. The World Health Organization Quality of Life Instruments (WHOQOL-BREF), a generic health- related questionnaire developed by the WHOQOL group was selected to quantify the HRQOL of ESRD patients [25]. The Nepali version of the WHOQOL-BREF questionnaire has been used in cross-cultural settings by Giri et al. [26] and Mishra et al. [27]. The WHOQOL-BREF consists of 26 items and provides a profile of scores on four dimensions of quality of life: physical health, psychological health, social relationships and environment health domain as well as two generic questions on overall perception of QOL and general health. The scores in the four domains were the outcome variables while all other variables were considered independent variables. Higher scores represented better quality of life. The scores on the 26^−^ item questions were measured on a scale of 4–20 [25].

The socio-demographic information included age (continuous; 20–30, 31–40, 41–50, > 50 years), sex (male and female), residence (urban and rural), socio-economic status (lower, middle and upper), ethnic group (*Brahmin/Chhetri, Janajati* and others), marital status (ever married, unmarried), employment (unemployed and employed), education (illiterate, up to 10 years of schooling and higher education), food habit (vegetarian and non-vegetarian) and perceived family support (full, partial and no support). Residence was considered as rural if the participant belonged to rural municipality and urban if residing in municipality, sub-metropolitan city and metropolitan city. The socio-economic status of the study participants was measured using “Kuppuswamy’s socio-economic status scale for Nepal [28]. It is measured on the basis of literacy level, type of occupation and family income level per month. This tool was developed in India in 2009 in which socioeconomic status is identified based on total score: 26–29 score for upper level, (b) 16–25 score for upper middle, (c) 11–15 score for lower middle, (d) 5–10 score for upper lower and (e) < 5 score for lower level. In this study, the upper middle and lower middle were merged as the middle while the upper lower and lower were merged as the lower. Similarly, clinical information related to ESRD included donor for transplant, duration of dialysis or transplant and presence of comorbidity (hypertension, diabetes, nephritic syndrome, others).

### Data collection procedure

Data were collected by face to face interviews. Four research assistants (public health undergraduates) along with the first author were involved in the interviews at the study sites. The research assistants were personally briefed and trained by the first author beforehand. Pre-testing of tools was carried out among five dialysis and five transplant recipients attending Bir Hospital located in Kathmandu. Based on pre-testing, minor changes in wording and sequence of the questions were made. Cronbach’s alpha test was computed for each domain and the value ranged from 0.60 to 0.86 indicating good internal consistency. Ethical approval for the study was obtained from the Ethical Review Board of the Nepal Health Research Council (Reference number: 389/2016), and administrative approval was taken from data collection sites. Prior written informed consent was obtained from the participants or their primary relatives, whichever applicable.

### Data analysis

The data were entered in EpiData version 3.1 and the data were transported to IBM SPSS version 21.0 for analysis. Descriptive analysis included calculation of frequency, percentage, mean and median for presentation of socio-demographic, ESRD-related and WHOQOL-BREF scores. The chi-square test was used to assess differences in categorical variables while the Mann-Whitney U test and Kruskal-Wallis one-way analysis of variance tests were used to compare QOL across socio-demographic and clinical characteristics since the QOL score was not normally distributed. The level of significance was maintained at 5% with *p* < 0.05 considered statistically significant.

## Results

### Demographic characteristics of the study participants

Among 161 participants, 92 (57.1%) were kidney transplant recipients and the remaining 69 (42.9%) were haemodialysis patients. In this study, 54.7% were 18–40 years of age, 75.2% were male and 47.3% belonged to the *Aadibasi/Janajati* ethnic group. The mean age (±SD) of the study participants was 40.66 ± 12.02 years. Most of the participants (58.4%) belonged to middle socioeconomic status, 12.4% were illiterate and 50.3% were unemployed. Nearly two-thirds (65.8%) of the participants received full support from their family in care and psychological support, while one in ten participants did not receive any support from their family (Table 1).

**Table 1:** Socio-demographic characteristics of the study participants (*n* = 161)

Characteristics	Total (*n* = 161) n (%)	Dialysis (*n* = 69) n (%)	Transplant (*n* = 92) n (%)	*P*-value
Age (years)	(40.66 ± 12.02)	43.57 ± 13.02	38.47 ± 10.77	< 0.01
20–30	36 (22.4)	14 (20.3)	22 (23.9)	
31–40	52 (32.3)	17 (24.6)	35 (38.0)	
41–50	40 (24.8)	15 (21.7)	25 (27.2)	
> 50	33 (20.5)	23 (33.3)	10 (10.9)	
Sex				0.958
Male	121 (75.2)	52 (75.4)	69 (75.0)	
Female	40 (24.8)	17 (24.6)	23 (25.0)	
Residence				0.234
Urban	124 (77.0)	50 (72.5)	74 (80.4)	
Rural	37 (23.0)	19 (27.5)	18 (19.6)	
Socioeconomic status				0.061
Lower	57 (35.4)	31 (44.9)	26 (28.3)	
Middle	94 (58.4)	33 (47.8)	61 (66.3)	
Upper	10 (6.2)	5 (7.2)	5 (5.4)	
Ethnic group				0.203
Brahmin/ Chhetri	57 (35.4)	21 (30.4)	36 (39.1)	
Aadibashi/Janajati	76 (47.2)	32 (46.4)	44 (47.8)	
Others	28 (17.4)	16 (23.2)	12 (13.0)	
Marital status				0.652
Unmarried	11 (6.8)	4 (5.8)	7 (7.6)	
Ever married	150 (93.2)	65 (94.2)	85 (92.4)	
Employment				0.020
Employed	80 (49.7)	27 (39.1)	53 (57.6)	
Unemployed	81 (50.3)	42 (60.1)	39 (42.4)	
Education				0.021
Illiterate	20 (12.4)	14 (20.3)	6 (6.5)	
Up to 10 years of schooling	96 (59.6)	40 (58.0)	56 (60.9)	
Higher	45 (28.0)	15 (21.7)	30 (32.6)	
Food habit				0.719
Vegetarian	6 (3.7)	3 (3.3)	3 (4.7)	
Non vegetarian	155 (96.3)	66 (96.7)	89 (95.3)	
Family support				0.513
Full	106 (65.8)	42 (60.9)	64 (69.6)	
Partial	39 (24.2)	19 (27.5)	20 (21.7)	
No support	16 (9.9)	8 (11.6)	8 (8.7)	

The age of the patients in the dialysis group was significantly higher than that in the transplant group (*p* < 0.01), and there was a significant difference in education status (*p* = 0.021) and employment status (*p* = 0.020) across the two patient groups. However, there was no significant difference according to sex, residence, socio-economic status, ethnic group, marital status or perceived family support in the patient groups (Table 1). Patients with renal transplant received kidney donation mainly from their parents (31.5%), spouse (30.4%) and children (25.0%). The duration of renal replacement therapy was more than one year for 67.4% of the renal transplant recipients and 53.4% for the dialysis patients. Regarding comorbidities, 77.0% had hypertension, 20.5% had diabetes mellitus, 5% had nephrotic syndrome, and 6.2% had other comorbidities.

### WHOQOL-BREF scores of dialysis and renal transplant recipients

Table 2 shows the mean score for QOL in different domains of the WHOQOL-BREF. The highest mean score for QOL was found in the social relationship (13.58 ± 2.14) domain, and the lowest mean score was found in the environmental health domain (11.73 ± 1.89). Haemodialysis patients scored significantly lower than the transplant recipients in terms of physical (*p* < 0.001), psychological (p < 0.001), social relationship (*p* = 0.012) and environmental health (*p* = 0.004) domains. Similarly, the overall QOL score (*p* < 0.001), overall perception of quality of life, Q1 (*p* < 0.001) and overall perception of general health, Q2 (p < 0.001) were significantly lower in haemodialysis participants than in transplant recipients (p < 0.001).

**Table 2:** Mean domain score for haemodialysis and renal transplant recipients

Type of domain	Total (*n* = 161)	Dialysis (*n* = 69)	Transplant (n = 92)	*P*-value
Physical	12.03 ± 2.16	10.61 ± 1.99	13.09 ± 1.61	< 0.001
Psychological	12.38 ± 2.44	10.84 ± 1.95	13.53 ± 2.12	< 0.001
Social relationship	13.58 ± 2.14	13.15 ± 2.10	13.89 ± 2.13	0.012
Environment health	11.73 ± 1.89	11.25 ± 1.62	12.10 ± 2.00	0.004
Perception of quality of life	3.03 ± 0.90	2.42 ± 0.72	3.49 ± 0.73	< 0.001
Perception of general health	3.07 ± 0.94	2.51 ± 0.80	3.49 ± 0.81	< 0.001
Overall QOL score	12.43 ± 1.63	11.46 ± 1.35	13.15 ± 1.45	< 0.001

### Socio-demographic variables, ESRD characteristics and QOL score

**Fig. 1:**
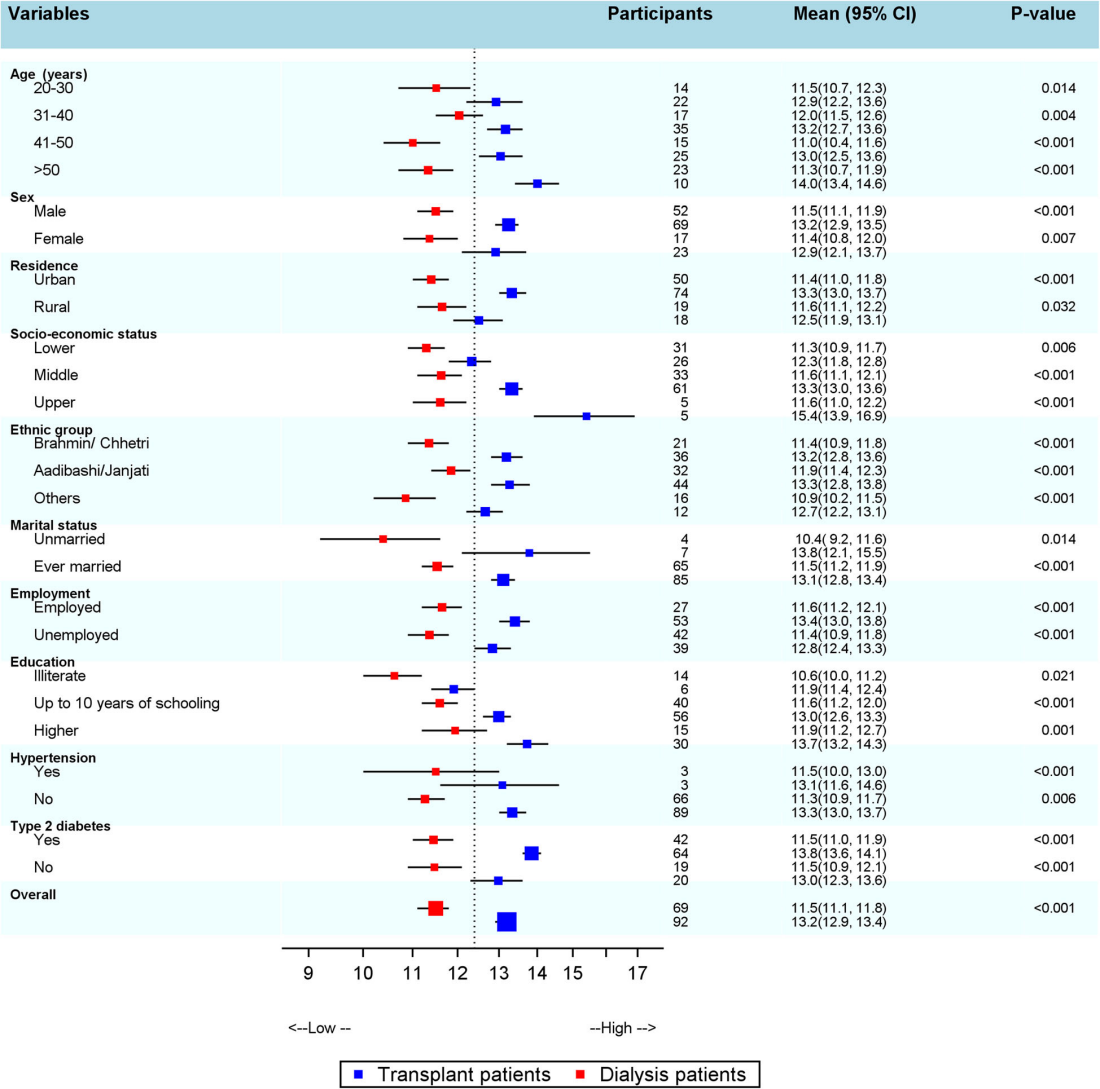
QOL score across socio-demographic and ESRD characteristics. The error bars show the mean (95% CI) QOL for dialysis (red ) and renal transplant patients (blue ). The size of the square is inversely proportional to the variance of the mean. The dotted vertical line shows the average QOL of the participants. The *P*-value shows the test for the difference in QOL between haemodialysis and transplant recipient patients across the socio demographic and ESRD variables. The individual estimates are shown in Additional file 1

The mean QOL scores across socio-demographic and ESRD characteristics are presented in Fig. 1. Ethnicity (*p* = 0.020), socio-economic status (*p* < 0.001), employment (*p* = 0.009) and education (p < 0.001) of the ESRD patients were significantly associated with the overall QOL (Additional file 1). Among ethnic groups, *Aadibasi/Janajati* had higher QOL than *Brahmin/Chhetri* and other ethnic groups. The QOL increased with the increase in socio-economic gradient and educational status. Age, sex, residence, marital status, hypertension and diabetes status were however not significantly associated (*p* > 0.05) with QOL among ESRD patients.

The mean differences in QOL scores were also assessed across haemodialysis and transplant recipients. The findings showed that there was a statistically significant difference in QOL among haemodialysis and transplant recipients across socio-demographic and clinical characteristics (*p* < 0.05) with higher QOL scores in transplant recipients than in haemodialysis patients (Fig. 1, & Additional file 1). Among haemodialysis patients, there was significant difference in QOL across educational status (*p* = 0.012), where those with higher educational status had a higher QOL. In the case of transplant recipients, urban residence (*p* = 0.023), higher socio-economic status (*p* < 0.001), higher education (*p* = 0.004) and diabetes status (*p* = 0.010) was significantly associated with better QOL (Fig. 1, & Additional file 1). Similarly, higher socio-economic status and higher educational status of the study participants were significantly associated with better QOL in all domains (Additional file 1).

## Discussion

Quality of life is being increasingly recognized as one of the key outcome parameters in any medical and interventional treatment. To our knowledge, this is one of the few studies to report a comparison of QOL between haemodialysis and renal transplant patients in Nepal. The study findings revealed significantly higher QOL scores in transplant recipients than in haemodialysis patients across all domains: physical, psychological, social relationships and environmental health. Similarly, the overall perception regarding QOL and general health was also significantly higher in transplant recipients than in haemodialysis patients. A study from Nepal using the same tool in 2011 showed significantly higher QOL among transplant recipients compared to haemodialysis patients in the physical, psychological and social relationship but not in the environment health domain [29]. Previous studies conducted elsewhere have also shown impaired QOL in haemodialysis patients as compared to transplant recipients [8, 30–34]. Although our study findings reinforce renal transplant as an effective RRT for improving QOL among people with ESRD, the health system in Nepal faces limited and inequitable access to transplant services. Furthermore, few nephrologists, long waiting times to receive transplant services and inadequate financial support add much to worry for people with ESRD [35].

The lower QOL in the physical domain in haemodialysis patients than in transplant recipients can be attributed to physical pain, weakness, insomnia and hindrance to daily activities as identified in previous studies [36, 37]. An earlier study from Nepal also showed an increased duration of haemodialysis as a negative predictor of QOL [38]. Similarly, the reason for renal transplant recipients having a higher QOL in the psychological domain might be due to a decrease in mental burden resulting from having to visit health facilities for frequent dialysis. Additionally, the increased self-esteem and improved perception regarding own health might have contributed to good mental wellbeing among renal transplant recipients [8]. Haemodialysis patients often face mental health problems due to their health conditions which might lead to a compromised QOL as identified by previous studies [37, 39, 40]. Renal transplant recipients had a higher QOL in the social domain than the haemodialysis patients in our study. This might be due to improved health including sexual relationships, and more leisure time, allowing them to network with their family and friends. Expensive treatment mainly drugs, difficulty in transportation during follow up and safety related issues might be contributing factors to the lower QOL score in the environmental domain among haemodialysis patients as compared to transplant recipients. The government pays hospitals a fixed amount for providing haemodialysis and transplant services for the destitute through its impoverished citizen fund established in 201 6[24, 35]. However, in the absence of renal registry and a fully viable health insurance system to cover expenses associated with the treatment, patients with ESRD face a major setback in living a quality life [35, 41]. Importantly, the government subsidy through the impoverished citizen fund does not cover all treatment expenses and moreover, there is a low enrollment and limited ceiling in benefit package (NPR 100,000) in health insurance [42]. In our study, even after transplant, QOL in the environmental health domain was found to be comparatively lower than that in other domains in renal transplant recipients. A social heath protection mechanism is thus imperative to address the financial barriers not only in seeking transplant services, but also saving people with chronic diseases in falling into the poverty trap.

In this study, socio-economic status was positively associated with the overall QOL score among ESRD patients. Among patient groups, there was a significant difference in QOL across socio-economic status in transplant recipients. As people with higher socio-economic status are in a better position to pay for treatment expenses, it might have resulted in higher QOL. However, this was not the case among haemodialysis patients which indicates that haemodialysis patients have a poor QOL regardless of their socio-economic status.

Employment status was associated with overall QOL among ESRD patients. Nepal has a large informal economy, a quarter of the population under the poverty line, and the majority of the population under poverty either sell their assets for paying for health care especially for the treatment of chronic diseases or simply stop their treatment [43, 44]. Those employed are more likely to be in a better paying capacity for their health care than the unemployed and face less financial catastrophe and possibly higher QOL.

This study has few strengths and limitations. In this study, WHOQOL-BREF tool was used to assess QOL which has been extensively used across different segments of the population in Nepal and globally, which makes it relevant for this study. One of the limitations of this study was that it sought to compare the HRQOL between only two categories of patients i.e. haemodialysis and transplant recipients and not with peritoneal dialysis and healthy populations, and such comparisons were done in a small sample of the population with differences in patient characteristics such as in age, education and employment. Moreover, the study might have encountered respondent bias due to subjective response and interviewer bias due to the first author’s involvement in interviewing the study participants. Additionally, the study participants were mostly male in this study which might be due to the higher burden of CKD in male than female in Nepal as per the national study [45]. Moreover, this might also indicate inequity in service utilization for renal replacement therapy [13]. Further studies employing a large sample and qualitative study to explore the in-depth experience of people with ESRD might help to generate more robust evidence regarding QOL in this population. Despite limitations, this study provides a comparative situation of QOL faced by haemodialysis and transplant recipients and the evidence could be useful for policy makers, program managers and other stakeholders for developing an effective response towards improving the health conditions of people with ESRD. Considering the increasing burden of CKD as well as other non-communicable diseases in Nepal, it would be effective to design interventions for the reduction of behavioral, biological and environmental risk factors associated with chronic disease outcomes.

## Conclusion

In summary, the QOL scores of renal transplant recipients were significantly better than those of haemodialysis patients in all four domains of the WHOQOL-BREF. Ethnicity, socio-economic status, educational status and employment status were significantly associated with the overall QOL in ESRD patients. Across patient groups, QOL improved significantly with an increase in socio-economic status in transplant recipients while socioeconomic status was not associated with QOL in haemodialysis patients. Renal transplant services should be encouraged for people with ESRD with strong social health security mechanism, as renal transplant recipients have a higher QOL than haemodialysis patients.

## Supplementary information

**Additional file 1.**

## Data Availability

All data related to this study are included in the manuscript and additional file.

## Abbreviations

CI: Confidence interval
CKD: chronic kidney diseases
ESRD: End stage renal disease
HRQOL: Health related quality of life
IOM: Institute of Medicine
MoHP: Ministry of Health and Population
NPHL: National Public Health Laboratory
QOL: Quality of life
RRT: Renal replacement therapy
SD: Standard deviation
TUTH: Tribhuvan University Teaching Hospital
WHO: World Health Organization
