# Supplementary material for: Health related quality of life among haemodialysis and kidney transplant recipients from Nepal: a cross sectional study using WHOQOL-BREF
Source: BMC Nephrol. 2020 Oct 12;21:433. doi: 10.1186/s12882-020-02085-0 (PMC7552453; doi:10.1186/s12882-020-02085-0)
Supplement: Supplementary file 1 — Additional file 1. [file 12882_2020_2085_MOESM1_ESM.docx]

**Additional File 1**

**Health related quality of life among haemodialysis and kidney transplant recipients from Nepal: a cross sectional study using WHOQOL-BREF**

Kamal Ranabhat^1,2^, Pratik Khanal^2⃰^, Shiva Raj Mishra^3,^ Anu Khanal^2^, Sangita Tripathi^2^, Mahesh Raj Sigdel^2^

1. Department of Health Services, Ministry of Health and Population, Kathmandu, Nepal
2. Institute of Medicine, Tribhuvan University, Kathmandu, Nepal
3. World Heart Federation, Geneva, Switzerland

⃰Corresponding author: Pratik Khanal, [pratikkhanal@iom.edu.np](mailto:pratikkhanal@iom.edu.np)

Additional Table 1: QOL score across socio demographic and ESRD characteristics

Additional Table 2: Socio-demographic, ESRD characteristics and domain wise QOL score

**Additional Table 1: QOL score across socio-demographic and ESRD characteristics**

| **Variables** | **Categories** | **Dialysis patients (n=69)** | **Transplant patients (n=92)** | **P^α^ value** | **Total (n=161)** | **P^β^ value** |
| --- | --- | --- | --- | --- | --- | --- |
|  |  | Mean (SD) | Mean (SD) |  | Mean (SD) |  |
| **Age (years)** | 20-30 | 11.51 (1.48) | 12.92 (1.73) | 0.014⃰ | 12.37 (1.75) | 0.167 |
|  | 31-40 | 12.03 (1.20) | 13.16 (1.30) | 0.004⃰ | 12.79 (1.37) |  |
|  | 41-50 | 11.00 (1.09) | 13.04 (1.50) | <0.001⃰ | 12.27 (1.68) |  |
|  | >50 | 11.33 (1.46) | 14.00 (0.99) | <0.001⃰ | 12.43 (1.64) |  |
|  | P^β^ value | 0.143 | 0.113 |  |  |  |
| **Sex** | Male | 11.50 (1.38) | 13.24 (1.26) | <0.001⃰ | 12.50 (1.57) | 0.238 |
|  | Female | 11.36 (1.28) | 12.91 (1.93) | 0.007⃰ | 12.25 (1.84) |  |
|  | P^β^ value | 0.656 | 0.267 |  |  |  |
| **Residence** | Urban | 11.40 (1.41) | 13.32 (1.45) | <0.001⃰ | 12.55 (1.71) | 0.081 |
|  | Rural | 11.64 (1.19) | 12.50 (1.30) | 0.032⃰ | 12.06 (1.30) |  |
|  | P^β^ value | 0.460 | 0.023⃰ |  |  |  |
| **SES** |  |  |  |  |  |  |
|  | Lower | 11.29 (1.22) | 12.33 (1.28) | 0.006⃰ | 11.76 (1.34) | <0.001⃰ |
|  | Middle | 11.62 (1.54) | 13.33 (1.26) | <0.001⃰ | 12.73 (1.58) |  |
|  | Upper | 11.60 (0.71) | 15.40 (1.66) | <0.001⃰ | 13.50 (2.33) |  |
|  | P^β^ value | 0.587 | <0.001⃰ |  |  |  |
| **Ethnic group** | Brahmin/ Chhetri | 11.35 (1.12) | 13.19 (1.31) | <0.001⃰ | 12.51 (1.53) | 0.020⃰ |
|  | Aadibashi/Janjati | 11.85 (1.43) | 13.27 (1.67) | <0.001⃰ | 12.67 (1.72) |  |
|  | Others | 10.85 (1.28) | 12.66 (0.82) | <0.001⃰ | 11.63 (1.42) |  |
|  | P^β^ value | 0.054 | 0.327 |  |  |  |
| **Marital status** | Unmarried | 10.39 (1.25) | 13.79 (2.25) | 0.014⃰ | 12.55 (2.54) | 0.835 |
|  | Ever married | 11.53 (1.34) | 13.11 (1.37) | <0.001⃰ | 12.43 (1.56) |  |
|  | P^β^ value | 0.147 | 0.167 |  |  |  |
| **Employment** | Employed | 11.64 (1.29) | 13.40 (1.45) | <0.001⃰ | 12.81 (1.62) | 0.009⃰ |
|  | Unemployed | 11.36 (1.39) | 12.83 (1.40) | <0.001⃰ | 12.07 (1.57) |  |
|  | P^β^ value | 0.393 | 0.110 |  |  |  |
| **Education** | Illiterate | 10.62 (1.09) | 11.91 (0.58) | 0.021⃰ | 11.01 (1.13) | <0.001⃰ |
|  | Up to 10 years of schooling | 11.59 (1.27) | 12.99 (1.31) | <0.001⃰ | 12.40 (1.06) |  |
|  | Higher | 11.94 (1.51) | 13.73 (1.61) | 0.001⃰ | 13.13 (1.78) |  |
|  | P^β^ value | 0.012⃰ | 0.004⃰ |  |  |  |
| **HTN** | Yes | 11.50 (1.29) | 13.08 (1.35) | <0.001⃰ | 12.33 (1.54) | 0.188 |
|  | No | 11.27 (1.72) | 13.34 (1.69) | 0.006⃰ | 12.78 (1.92) |  |
|  | P^β^ value | 0.603 | 0.646 |  |  |  |
| **DM** | Yes | 11.45 (1.52) | 13.84 (1.11) | <0.001⃰ | 12.82 (1.75) | 0.080 |
|  | No | 11.47 (1.32) | 12.98 (1.48) | <0.001⃰ | 12.33 (1.60) |  |
|  | P^β^ value | 0.864 | 0.010⃰ |  |  |  |

Notes. P^α^ -value shows the test for the difference in QOL between dialysis and transplant participants across socio-demographic and ESRD characteristics; P^β^ -value shows the test for difference in QOL across socio-demographic and ESRD characteristics for dialysis (n=69), transplant (n=92) and total participants (n=161). See methods section for use of individual tests in these analyses; SES: socio-economic status

**Additional Table 2: Socio-demographic, ESRD characteristics and domain wise QOL score**

| **Characteristics** | **Categories** | **Total ESRD patients (n=161)** | | | |
| --- | --- | --- | --- | --- | --- |
|  |  | **PHY** | **PSY** | **SOC** | **ENV** |
| **Age (years)** | 20-30 | 12.30 (2.14) | 11.85 (2.46) | 13.63 (2.12) | 11.71 (2.20) |
|  | 31-40 | 12.59 (1.92) | 12.78 (2.18) | 14.28 (1.84) | 11.50 (1.94) |
|  | 41-50 | 12.13 (1.98) | 12.65 (2.30) | 12.50 (2.59) | 11.81 (1.53) |
|  | >50 | 10.75 (2.34) | 12.00 (2.91) | 13.74 (1.51) | 12.06 (1.90) |
|  | P-value | 0.001⃰ | 0.225 | 0.001⃰ | 0.608 |
| **Sex** | Male | 12.14 (2.11) | 12.35 (2.53) | 13.73 (2.05) | 11.76 (1.75) |
|  | Female | 11.73 (2.32) | 12.47 (2.19) | 13.13(2.39) | 11.68 (2.30) |
|  | P-value | 0.302 | 0.799 | 0.128 | 0.806 |
| **Residence** | Urban | 12.08 (2.16) | 12.45 (2.54) | 13.65 (2.17) | 12.00 (1.93) |
|  | Rural | 11.88 (2.20) | 12.14 (2.12) | 13.37 (2.07) | 10.85 (1.48) |
|  | P-value | 0.612 | 0.504 | 0.494 | 0.001⃰ |
| **SES** | Lower | 11.52 (1.92) | 11.44 (2.03) | 12.94 (2.54) | 11.15 (1.45) |
|  | Middle | 12.30 (2.18) | 12.87 (2.45) | 13.89 (1.85) | 11.86 (1.84) |
|  | Upper | 12.46 (2.95) | 13.20 (3.12) | 14.40 (1.38) | 13.95 (2.80) |
|  | P-value | 0.078⃰ | 0.001⃰ | 0.013⃰ | <0.001⃰ |
| **Ethnic group** | Brahmin/ Chhetri | 12.28 (2.04) | 12.42 (2.45) | 13.52 (2.29) | 11.83 (1.79) |
|  | Aadibashi/Janjati | 12.08 (2.31) | 12.75 (2.47) | 13.86 (1.77) | 12.01 (1.95) |
|  | Others | 11.43 (1.94) | 11.31 (2.13) | 12.95 (2.66) | 10.82 (1.70) |
|  | P-value | 0.229 | 0.028⃰ | 0.155 | 0.015⃰ |
| **Marital status** | Unmarried | 12.52 (2.80) | 11.45 (3.41) | 13.45 (1.83) | 12.77 (3.17) |
|  | Ever married | 12.00 (2.12) | 12.45 (2.36) | 13.59 (2.17) | 11.66 (1.76) |
|  | P-value | 0.444 | 0.194 | 0.839 | 0.061 |
| **Employment** | Employed | 12.34 (2.09) | 13.04 (2.40) | 13.82 (2.03) | 12.03 (2.14) |
|  | Unemployed | 11.73 (2.21) | 11.73 (2.32) | 13.35 (2.25) | 11.46 (1.58) |
|  | P-value | 0.073 | 0.001⃰ | 0.168 | 0.057 |
| **Education** | Illiterate | 10.29 (1.95) | 10.73 (2.03) | 12.53 (1.75) | 10.48 (1.61) |
|  | Up to 10 years of schooling | 12.15 (2.02) | 12.36 (2.28) | 13.57 (2.30) | 11.53 (1.50) |
|  | Higher | 12.56 (2.20) | 13.16 (2.63) | 14.07 (1.81) | 12.74 (2.29) |
|  | P-value | <0.001⃰ | 0.001⃰ | 0.027⃰ | <0.001⃰ |
| **HTN** | Yes | 11.85 (2.12) | 12.27 (2.29) | 13.51 (2.18) | 11.70 (1.71) |
|  | No | 12.66 (2.21) | 12.76 (2.92) | 13.84 (2.05) | 11.86 (2.43) |
|  | P-value | 0.044⃰ | 0.288 | 0.410 | 0.647 |
| **DM** | Yes | 12.07 (2.70) | 13.01 (2.67) | 14.18 (1.48) | 12.03 (1.75) |
|  | No | 12.03 (2.02) | 12.22 (2.37) | 13.43 (2.26) | 11.66 (1.93) |
|  | P-value | 0.920 | 0.098 | 0.071 | 0.324 |

Note: PHY: Physical; PSY: Psychological; SOC: Social; ENV: Environment; ⃰ statistically significant at p<0.05; SES: socio-economic status
